# *In Vitro* Aggregation Behavior of a Non-Amyloidogenic *λ* Light Chain Dimer Deriving from U266 Multiple Myeloma Cells

**DOI:** 10.1371/journal.pone.0033372

**Published:** 2012-03-14

**Authors:** Paolo Arosio, Marta Owczarz, Thomas Müller-Späth, Paola Rognoni, Marten Beeg, Hua Wu, Mario Salmona, Massimo Morbidelli

**Affiliations:** 1 Institute for Chemical and Bioengineering, Department of Chemistry and Applied Biosciences, ETH Zurich, Zurich, Switzerland; 2 Department of Molecular Biochemistry and Pharmacology, Istituto di Ricerche Farmacologiche “Mario Negri,” Milan, Italy; 3 Amyloid Research and Treatment Center, Foundation IRCCS Policlinico San Matteo, University of Pavia, Pavia, Italy; Consejo Superior de Investigaciones Cientificas, Spain

## Abstract

Excessive production of monoclonal light chains due to multiple myeloma can induce aggregation-related disorders, such as light chain amyloidosis (AL) and light chain deposition diseases (LCDD). In this work, we produce a non-amyloidogenic IgE *λ* light chain dimer from human mammalian cells U266, which originated from a patient suffering from multiple myeloma, and we investigate the effect of several physicochemical parameters on the *in vitro* stability of this protein. The dimer is stable in physiological conditions and aggregation is observed only when strong denaturating conditions are applied (acidic pH with salt at large concentration or heating at melting temperature *T_m_* at pH 7.4). The produced aggregates are spherical, amorphous oligomers. Despite the larger β-sheet content of such oligomers with respect to the native state, they do not bind Congo Red or ThT. The impossibility to obtain fibrils from the light chain dimer suggests that the occurrence of amyloidosis in patients requires the presence of the light chain fragment in the monomer form, while dimer can form only amorphous oligomers or amorphous deposits. No aggregation is observed after denaturant addition at pH 7.4 or at pH 2.0 with low salt concentration, indicating that not a generic unfolding but specific conformational changes are necessary to trigger aggregation. A specific anion effect in increasing the aggregation rate at pH 2.0 is observed according to the following order: SO_4_^−^≫Cl^−^>H_2_PO_4_^−^, confirming the peculiar role of sulfate in promoting protein aggregation. It is found that, at least for the investigated case, the mechanism of the sulfate effect is related to protein secondary structure changes induced by anion binding.

## Introduction

The aggregation stability of the immunoglobulin light chain fragments is involved in several disorders related to the abnormal proliferation of bone marrow monoclonal plasma B cells and the subsequent excessive production of monoclonal light chains. 10–15% of the patients affected by multiple myeloma and with large light chain serum concentration are subsequently affected by the aggregation-related diseases [1], such as light chain amyloidosis (AL) [2], light chain deposition diseases (LCDD) [3], and cast nephropathy [4]. Light chain amyloidosis (AL) is the most common form of sporadic systemic amyloidosis [5], [6], characterized by deposition of insoluble amyloid fibrils in organs such as kidney, heart and liver, which leads to organ failure; it can act also on peripheral nerve, gastrointestinal track and lungs [7]. In opposition to the fibrillar structures encountered in AL, in LCDD the aggregates are amorphous and granular. The basement membrane of kidney is the main target organ, although also heart and liver may be affected [8]. Typically, only one of the two forms of disease occurs in patients [9].

A great challenge in understanding the cause and mechanism of aggregation is given by the extremely large number of the possible mutated variant sequences involved. In fact, each patient produces antibodies which have undergone antigen-driven selection. Therefore, the heterogeneity of possible diseases and related protein aggregates are due both to fragment primary sequence and to environmental factors. Several works show that the *κ* and *λ* types of light chain dominate in LCDD and AL, respectively [10]. Despite such tentative of classification based on clinical analysis [11], [12], the factors determining whether or not some variants are pathological or lead to fibrillar aggregates or amorphous deposits are far from being understood.

In addition to protein structure, protein amount affects propensity to deposit: circulating disulfide-bound light chain dimers can interfere with normal clearance and metabolism, increasing their serum level [11]. Therefore, it is important to clarify the stability of the dimers and the relationship between dimer/monomer equilibrium and aggregation mechanism [13].

In this work, we investigate the effect of several physicochemical parameters on the *in vitro* aggregation propensity of a *λ* light chain IgE dimer. This protein was obtained from a human myeloma cell line U266, coming from a patient suffering from multiple myeloma in which neither amyloids nor amorphous deposits were detected [14]. The production of protein in a reasonable amount and in a reproducible way is often a challenge in protein aggregation studies. Commonly, light chain variants are produced recombinantly from bacteria [15] or extracted from urine [16]. Despite the larger effort demanded compared to the commonly employed bacteria systems, the production from eukaryotic cells allows avoiding re-folding steps commonly present in the production from bacteria. Moreover, in the case of glycosilated light chain variants the use of eukaryotic cells allows the production of proteins with a glycosylation pattern close to the physiological one. Although the production of light chain fragment and complete immunoglobulin by U266 cells has been already studied [17], [18], to our knowledge this is the first aggregation study on proteins obtained by such cell line.

We applied several biophysical techniques to investigate protein secondary structure stability and aggregation behavior. Protein secondary structure was characterized by spectroscopic techniques such as circular dichroism (CD), intrinsic tryptophan fluorescence and 8-anilino-1-naphthalenesulfonic acid (ANS) binding, while the aggregation was monitored by dynamic light scattering (DLS), thioflavin T (ThT) assay, size exclusion chromatography (SEC) and asymmetrical field flow fractionation (FFF). Aggregates morphology was investigated by Congo Red binding, thin film attenuated total reflectance fourier transform infrared spectroscopy (ATR-FTIR) and atomic force microscopy (AFM). The study shows the relationship between secondary structure and aggregation for the investigated λ light chain dimer and underlines the importance of environmental factors, particularly the kind and concentration of salt, on protein stability.

Moreover, we have successfully determined the original full-length sequence of the monoclonal light chain produced by the human U266 cell clone. This observation will enable us to make comparative analysis with other studies describing the features of immunoglobulin light chains with a known sequence.

## Results

### Protein characterization

**Figure 1:**
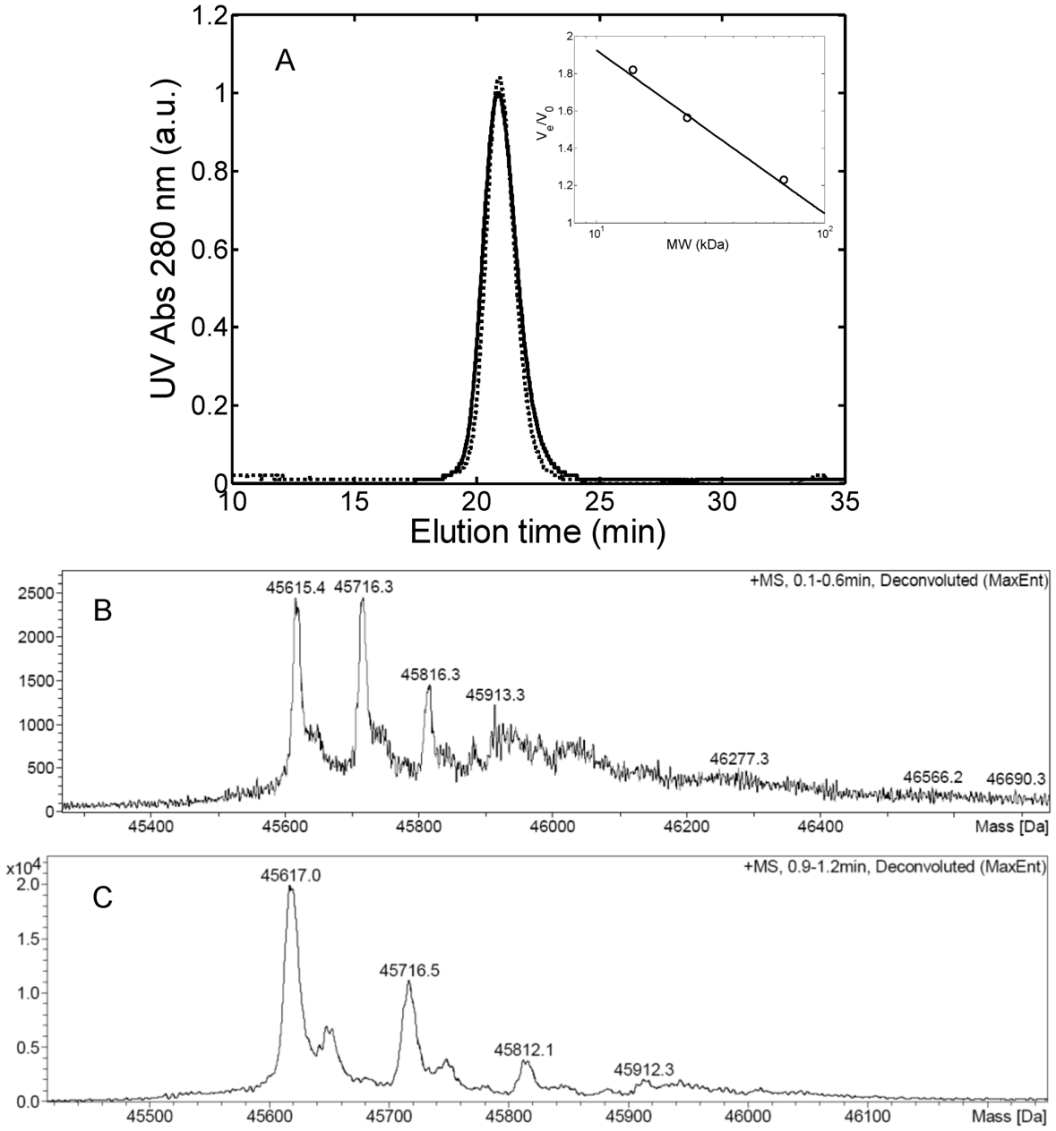
Protein characterization. (A) SEC chromatogram of 1 g/L light chain solution in 25 mM PBS buffer at pH 7.4 (―) and in 20 mM HCl at pH 2.0 (–). Insertion: calibration curve obtained with bovine serum albumin (60 kDa), chymotrypsinogen A (25 kDa) and lysozyme (14.5 kDa); (B) and (C) Mass spectroscopy analysis in 25 mM PBS buffer at pH 7.4 (B) and in 20 mM HCl at pH 2.0 (C).

Many domains (both single variable and complete constant- variable domains) have been found to form dimers in physiological and stable conditions [16]. Particularly, amyloidogenic light chains are commonly found to be in equilibrium between the dimer and the monomeric form in patients' urine [16]. After the production and purification step, the protein has been characterized in terms of monomer-dimer equilibrium at physiological and low pH. The SEC chromatograms reported in Figure 1A show a single, symmetric peak under both conditions. According to the column calibration curve, the elution volume of such a peak corresponds to a molecular weight of 42 kDa, indicating the presence of a dimer consisting of both variable and constant domain [1]. The linearity between the elution volume and the natural logarithm of the molecular weight applies well only for globular proteins and non-spherical macromolecules can deviate from such relationship [19]. Therefore, to get a more accurate evaluation of the molecular weight, mass spectroscopy (MS) analysis was performed. The results in Figure 1B and 1C show one single peak with molecular weight equal to 45.7 kDa for both conditions, in agreement with SEC analysis. It is worth noting that the results of SEC and MS are inconsistent with the western Blot and SDS-PAGE analysis (data not shown), which revealed two bands, at around 50 kDa and 25 kDa. This may arise from the denaturating conditions used in the electrophoresis analysis, which can induce breakage of the dimer disulphide-bond. Indeed, when SDS-Page was performed in the presence of a reducing agent, only the 25 kDa monomer band was detected. We can conclude that the produced light chains are composed of both constant and variable part and associate into dimers through covalent disulphide bonds, which can be broken under reducing conditions.

The nucleotide sequence of U266 derived IgE λ light chain has been characterized as described in Text S1. Figure S1 reports the U266 derived IgE λ light chain nucleotide sequence, whereas Figure S2 shows the alignment of the amino acid sequence with the germline donor IGVL2-8.

In addition, the pI of the protein was measured equal to 8.5–9.0 by isoelectric focusing (data not shown), in agreement with the theoretical pI (8.25), calculated using a dedicated tool available on the Expasy proteomic server website (www.expasy.org).

### Effect of environmental factors on structure stability and aggregation behavior

The effect of pH, denaturant and temperature on the structure stability of the light chain was investigated by spectroscopic techniques.

**Figure 2:**
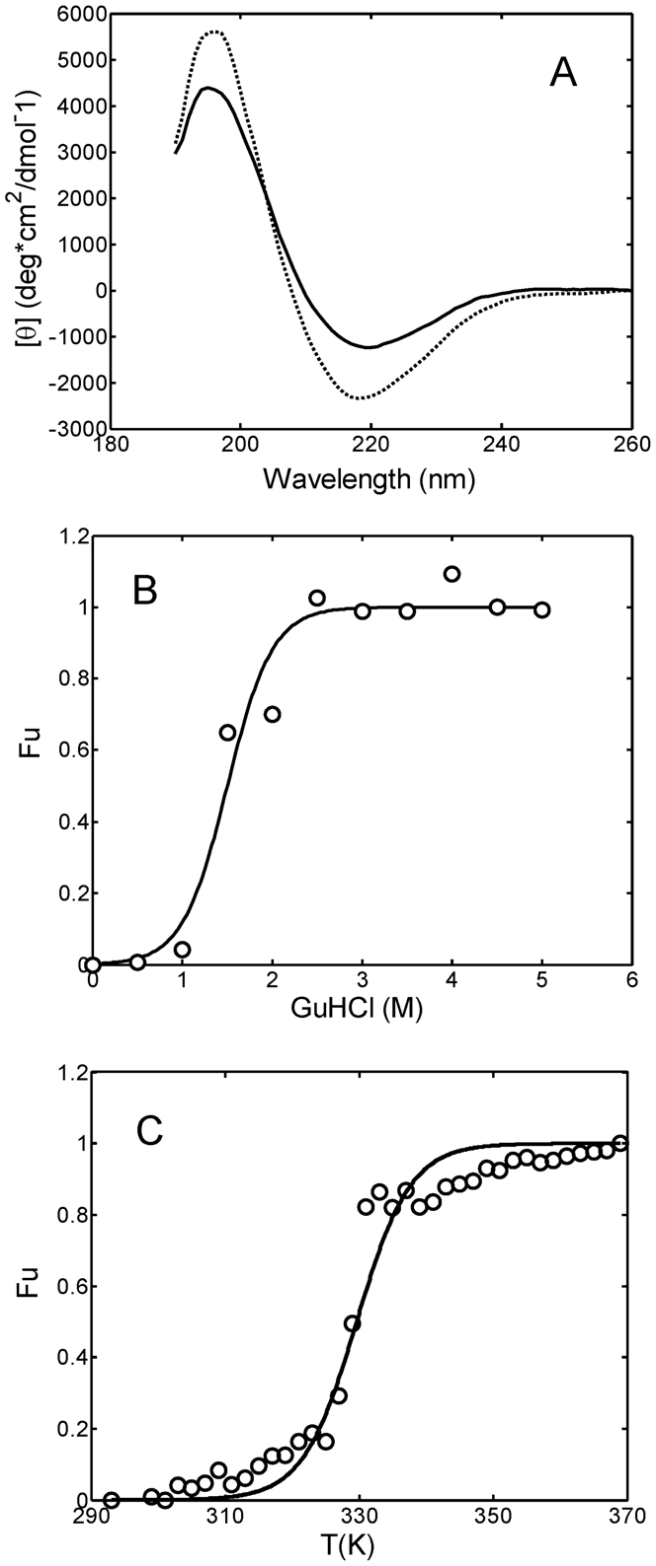
Light chain structural changes induced by pH, denaturant addition and temperature. (A) CD spectra for the 0.3 g/L light chain solution in 25 mM PBS at pH 7.4 (―) and in 20 mM HCl at pH 2.0 (–); (B) Fraction of unfolded protein as a function of guanidinium hydrochloride (GuHCl) concentration evaluated by intrinsic tryptophan fluorescence measurements (see Materials and Methods). The continuous line represents the interpolation of experimental data according to Eq. 1; (C) Fraction of unfolded protein as a function of temperature evaluated by CD measurements (see Materials and Methods). The continuous line corresponds to the interpolation of experimental data according to Eq.1.

In Figure 2A the far-UV CD spectra of the protein at pH 7.4 and pH 2.0 are reported. The spectrum at pH 7.4 shows a minimum at 220 nm characteristic of the Greek key β-barrel folding of the immunoglobulin fragment [20], [21]. The β-sheet structure content increases as the pH value decreases from 7.4 to 2.0 and the minimum shifts from 220 to 218 nm.

The unfolded protein fraction as a function of guanidinium hydrochloride (GuHCl) has been evaluated by intrinsic tryptophan fluorescence, while the temperature stability of the light chain was investigated by CD temperature-step measurements (see Materials and Methods for details). The GuHCl concentration required to unfold half of the protein (*F_u_* = 0.5) is equal to about 1.5 M (Figure 2B), while the melting temperature (*T_m_*) at physiological pH was estimated equal to 55°C (Figure 2C).

After characterizing the structural stability of the protein, the aggregation propensity of the light chain in several conditions was assessed by DLS. A summary of all the experimental runs is reported in Table 1, while the corresponding DLS data are shown in Figure S3. For the condition at low pH, the effect of biologically relevant salts, i.e. sodium chloride (NaCl), sodium phosphate (NaH_2_PO_4_) and sodium sulphate (Na_2_SO_4_), was considered.

**Table 1:** Summary of all investigated conditions. Protein concentration is 1 g/L in all cases.

Run	Buffer	pH	Salt	T	Aggregation
1	25 mM PBS	7.4	0.15 M NaCl	37°C	**no**
2	20 mM HCl	2.0	-	37°C	**no**
3	20 mM HCl	2.0	0.15 M NaCl	37°C	**no**
4	20 mM HCl	2.0	0.45 M NaCl	37°C	***yes***
5	20 mM HCl	2.0	0.15 M NaH_2_PO_4_	37°C	**no**
6	20 mM HCl	2.0	0.49 M NaH_2_PO_4_	37°C	***yes***
7	20 mM HCl	2.0	0.064 M Na_2_SO_4_	37°C	***yes***
8	20 mM HCl	2.0	0.15 M Na_2_SO_4_	37°C	***yes***
9	20 mM HCl	2.0	0.5 M Na_2_SO_4_	37°C	***yes***
10	20 mM HCl	2.0	0.45 M KCl	37°C	***yes***
11	25 mM PBS	7.4	-	55°C	***yes***
12	25 mM PBS	7.4	0.15 M NaCl + 1.3 to 1.5 M GnHCl	37°C	**no**
13	25 mM PBS	7.4	0.15 M Na_2_SO_4_ + 1.5 M GnHCl	37°C	**no**
14	25 mM PBS	7.4	0.15 M Na_2_SO_4_ + seeds	37°C	**no**

In physiological conditions, *i.e.*, 25 mM PBS with 0.15 M NaCl at pH 7.4 and 37°C (run 1 in Table 1), the protein solutions were stable for over five months. Also in 20 mM HCl solution at pH 2.0 with or without 0.15 M NaCl or 0.15 M NaH_2_PO_4_ no aggregation could be detected after several days of incubation (run 2, 3 and 5 in Table 1).

**Figure 3:**
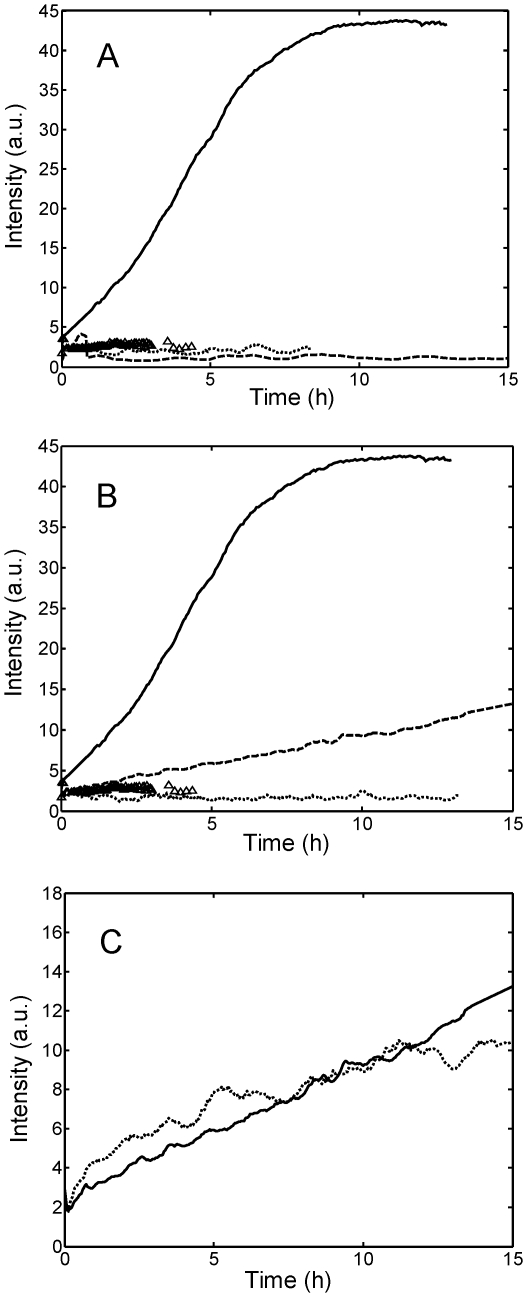
Light scattering intensities measured on-line by *in situ* DLS. (A) 1 g/L light chain solution in 20 mM HCl buffer at pH 2.0 without salt (▵), with 0.15 M NaCl (–), NaH_2_PO_4_ (…), Na_2_SO_4_ (―) (runs 3, 4, 6 and 9 in Table 1); (B) The same as (A) but salts are compared at similar ionic strength (see text): 0.45 M NaCl (–), 0.49 M NaH_2_PO_4_ (…), 0.15 M Na_2_SO_4_ (―) (runs 4, 6 and 8 in Table 1); (C) The same as (A) but for a solution in 20 mM HCl buffer at pH 2.0, with 0.45 M NaCl (―) and KCl (…) (runs 4 and 10 in Table 1).

The situation changed when the concentrations of NaCl and NaH_2_PO_4_ were increased to 0.45 M and 0.49 M, respectively (run 4 and 6 in Table 1) or when Na_2_SO_4_ was added (runs 7 to 9 in Table 1). The stability behavior for the different salts and different salt concentrations, followed by DLS, is shown in Figure 3. In Figure 3A, the results obtained using different salts but at the same constant concentration of 0.15 M are compared, while in Figure 3B salts are compared at similar ionic strength *I*, being *I* defined as 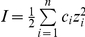, where *c_i_* is the concentration of ionic species and *z_i_* the corresponding valence (*I* is equal to 0.45 M for a 0.45 M NaCl and 0.49 M NaH_2_PO_4_ solution, and equal to 0.35 M for a 0.15 M Na_2_SO_4_ solution). In all cases where instability occurred, the scattered intensity increased almost linearly from the beginning of the incubation, without the lag phase typically encountered in amyloidogenic systems [22], indicating that the aggregate formation started immediately after incubation without a nucleation process. NaH_2_PO_4_ followed a behavior similar to NaCl, *i.e.*, aggregation was observed only at sufficiently large concentration values, but aggregation was slower with NaH_2_PO_4_ than with NaCl. Instead, Na_2_SO_4_ showed a peculiar effect in accelerating the aggregation at low pH, which occurred even at low salt concentration (64 mM, corresponding to an ionic strength of 0.15 M).

**Figure 4:**
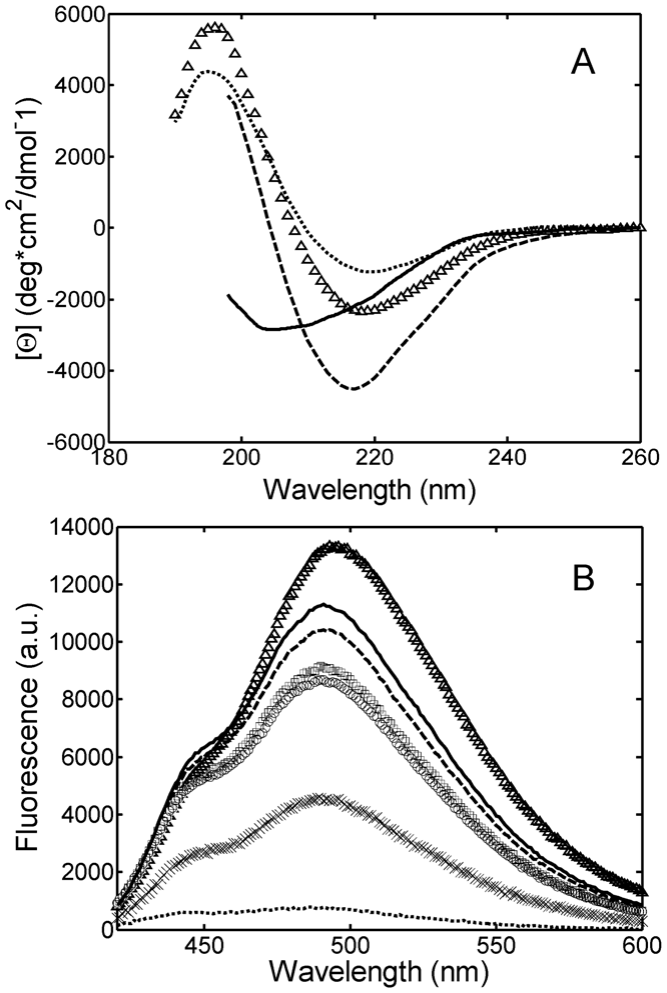
Light chain structural changes induced by salt addition monitored by CD (A) and ANS binding (B). Experiments were performed at 20°C for a 0.3 g/L protein solution in 25 mM PBS at pH 7.4 (…) and in 20 mM HCl buffer at pH 2.0 without salt (▵), with 0.15 M NaCl (–),0.15 M Na_2_SO_4_ (―), 0.45 M NaCl (○), 0.49 M NaH_2_PO_4_ (□) and 0.5 M Na_2_SO_4_ (×).

To investigate such peculiar effect, the initial light chain secondary structure in the presence of the sulfate and the chloride anion was characterized by CD spectroscopy and ANS binding, a method probing the solvent accessibility of hydrophobic patches. As shown in Figure 4A, with respect to the case in the absence of salt, in the presence of NaCl the minimum in the far-UV CD spectrum is decreased, indicating an increase of β-sheet structure; moreover, the minimum shifts from 218 to 217 nm. Such structural rearrangement is accompanied by a burying of hydrophobic patches, as indicated by the decrease of the maximum ANS fluorescence (Figure 4B) as a consequence of the reduced binding of the hydrophobic dye. In the presence of the sulfate anions, the light chain structure changes significantly: the far-UV CD spectrum shows a minimum at 204.6 nm, corresponding to a disordered structure (Figure 4A). In such a more open, disordered structure, with respect to the β-sheet, the hydrophobic patches expose more in the solvent. Indeed, the ANS binding in this case is larger than that in the presence of the chloride anion, as indicated by the increase in maximum ANS fluorescence (Figure 4B). It is likely that such significant structural change forms an intermediate more prone to aggregate. On the other hand, by increasing the salt concentration (by adding 0.45 M NaCl, 0.49 M NaH_2_PO_4_ and 0.5 M Na_2_SO_4_), the maximum ANS fluorescence decreases (Figure 4B), indicating less hydrophobic conformations. This is likely due to the additional effects induced at high salt concentration, which will be discussed in the Discussion section. Moreover, it is worth noticing that at 0.5 M Na_2_SO_4_ the fast aggregation rate affects the ANS measurements.

Since at low pH the protein is positively charged (pI = 8.5–9), only anions are expected to affect intra and intermolecular interactions. To verify the absence of the cation effect on aggregation, an additional experiment in 20 mM HCl at pH 2.0 with 0.45 M KCl was performed (run 11 in Table 1). The result is compared with that with 0.45 NaCl in Figure 4C, confirming the absence of the cation effect for the investigated salts.

After considering aggregation at low pH, we have also investigated temperature- and denaturant-induced aggregation at physiological pH. Incubation at the melting temperature *T_m_* = 55°C (run 11 in Table 1) caused aggregate formation after few hours. Instead, incubation in 25 mM PBS at pH = 7.4 with GuHCl in the concentration range from 1.3 to 1.5 M (run 12 and 13 in Table 1) could not induce any aggregation in the presence of either 0.15 M NaCl or 0.15 M Na_2_SO_4_. This result suggests that a generic partial protein unfolding is insufficient to promote aggregation, but specific unfolded configurations are necessary to induce aggregation.

### Aggregation pathway and aggregate morphology

**Figure 5:**
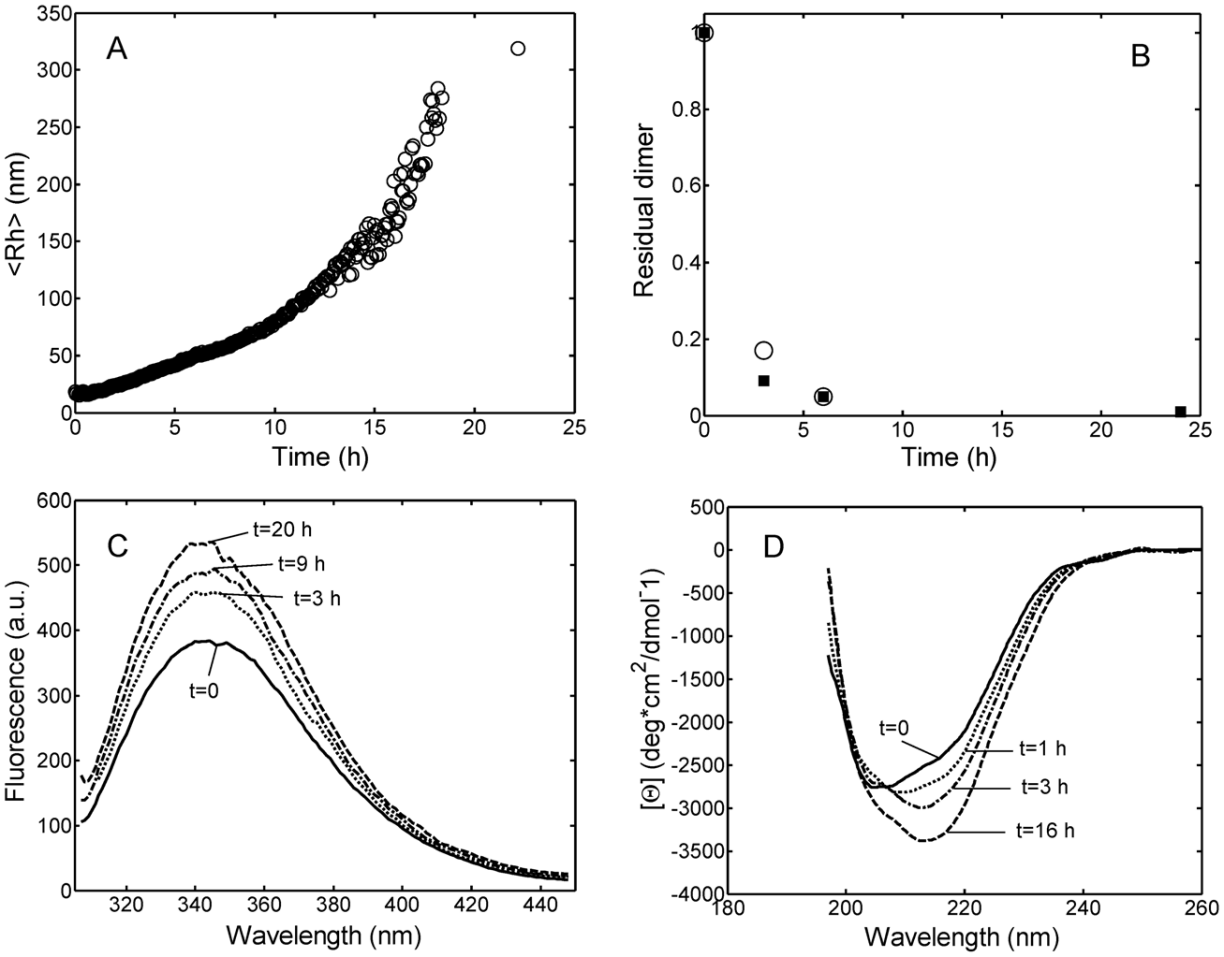
Aggregation kinetics of light chain solution in 20 mM HCl at pH 2.0, T = 37°C, with 0.15 M Na_2_SO_4_ (run 8 in **Table 1**). Time evolutions of (A) the average hydrodynamic radius *<R_h_>*, measured on-line by *in situ* DLS; (B) residual massive dimer fraction (*m*) evaluated by SEC (▪) and FFF (○); (C) Intrinsic tryptophan spectra; (D) CD spectra. Experiments were performed at a protein concentration of 1 g/L for (A) and (B) and 0.3 g/L for (C) and (D).

The aggregation pathway was investigated by several techniques taking as a reference condition run 8 in Table 1 (20 mM HCl buffer at pH 2.0 with 0.15 M Na_2_SO_4_, 37°C). In Figure 5A the time evolution of the hydrodynamic radius (*<R_h_>*) followed on-line by DLS is shown. As mentioned above, the aggregation induced by the sulfate addition occurs without lag-phase. The dimer conversion was measured by taking samples at different times during the aggregation and analyzing them off-line by SEC and FFF. The results obtained by the two techniques were consistent, showing a significant decrease of the dimer peak in the chromatograms over time (data not shown). From the value of the area under the peak, the amount of the residual massive dimer fraction has been evaluated and shown in Figure 5B. It is seen that the dimer conversion is almost completed already after 6 hours while aggregation is still on-going (Figure 5A). This implies that aggregation does not occur via dimer addition only but also among larger aggregates. However, larger aggregates could not be detected with neither of the two techniques mentioned above, probably because of interactions with the stationary phases of the two instruments.

The change of secondary structure during aggregation was followed by spectroscopic techniques (intrinsic tryptophan, CD and ThT fluorescence). The intrinsic tryptophan spectra showed an increase in the maximum fluorescence value over time (Figure 5C), indicating that, as a consequence of structural rearrangements, the tryptophan residues exposed during denaturation at low pH become less solvent-exposed during the aggregation. The change in the protein secondary structure along the aggregation is confirmed by CD spectroscopy, as shown in Figure 5D. The far-UV CD spectra show a progressive significant shift from a random-coil structure to a more ordered β-sheet structure, with the minimum shifted from 205 to 216 nm.

Despite the formation of more ordered β-sheet structure during aggregation, the aggregates showed low ThT signal increment with the respect to the starting value, indicating lower ThT binding (see Figure S4). The increase in solution turbidity was significant already after one day incubation. However, while the increase of the light scattering intensity was large, the increase of ThT absolute fluorescence values was small even after three weeks of incubation. After such period, aggregates were analyzed also with Congo Red test, the most common and efficient test applied to detect the presence of fibrils of light chain [23]. The result of the test was negative, confirming the absence of fibrillar structure (see Figure S5).

**Figure 6:**
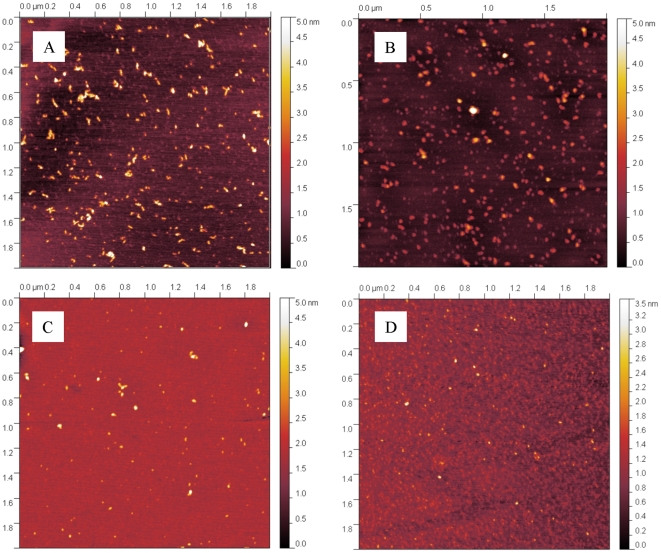
Aggregates morphology. AFM pictures of light chain aggregates obtained after one month incubation in 20 mM HCl at pH 2.0 with 0.45 M NaCl (A), with 0.15 M Na_2_SO_4_ (B) or with 0.49 M NaH_2_PO_4_ (C) and after two hours incubation in 25 mM PBS at pH 7.4, T = 55°C (D) (runs 4, 8, 6 and 11 in **Table 1**).

The aggregate morphology was further studied by AFM microscopy and hydrated thin film ATR-FTIR. In Figure 6 the pictures of samples taken after one month incubation in the conditions of runs 4, 6, 8 and 11 in Table 1 are shown. It can be seen that only spherical aggregates are visible for all the investigated conditions.

**Figure 7:**
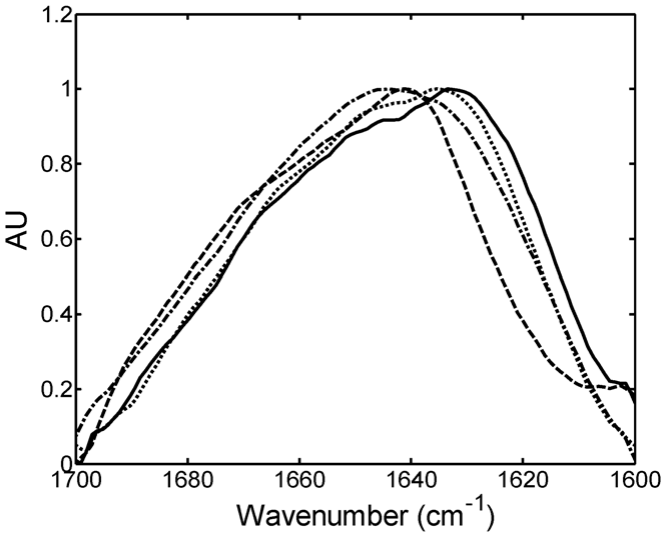
FTIR secondary structure analysis. Thin film ATR-FTIR spectra of native light chain dimer (–) and of light chain aggregates obtained after one month incubation in 20 mM HCl at pH 2.0 with 0.45 M NaCl (―), with 0.15 M Na_2_SO_4_ (…), or with 0.49 M NaH_2_PO_4_ (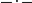) (runs 4, 8, and 6 in Table 1).

FTIR spectra of native state dimer and protein aggregates are reported in Figure 7, where the spectrum of the native dimer shows a maximum at 1641 cm^−1^, characteristic of the β-sheet structure of the immunoglobulin fragment and corresponding to the minimum at 220 nm in CD spectrum in Figure 2A. The spectrum shows also a significant presence of disordered, α-helix and turns/loops structure, corresponding to the area between 1647 and 1695 cm^−1^, in analogy to other spectra of variable domain reported in literature [24], [25], [26]. In the aggregate spectra the percentage of the area between wavenumbers 1615 and 1640 cm^−1^ with respect to the total area increases and the maximum shifts to 1635.4 cm^−1^. Such changes indicate rearrangement of the secondary structure inside the aggregates and, particularly, an increase of the β-sheet structure content with respect to the native state, in agreement with the intrinsic tryptophan fluorescence and the CD data shown in Figure 5C and 5D, respectively. Nevertheless, the disordered, α-helix and turns/loops structures are still significantly present, and the absence of the predominance of the β-sheet structure confirms again the non-amyloidogenic nature of the formed aggregates. The shift of the maximum is less pronounced in the case of NaH_2_PO_4_, due to the slower aggregation kinetics.

The ability of the obtained aggregates to seed aggregation was investigated by adding fragments of aggregates obtained in 20 mM HCl buffer at pH 2.0 with 0.5 M Na_2_SO_4_ to a 1 g/L light chain solution. The aggregates were broken by sonication as reported by Kim *et al.* [27] using two different sonication times of 0.5 and 5 minutes. The seeds were added at a concentration of 0.1 and 0.2 g/L to a solution at physiological pH with Na_2_SO_4_ (runs 14 in Table 1): no aggregation was observed after several months. The results indicate that amorphous aggregates alone are unable to induce aggregation under the conditions where the peculiar, partially unfolded, configuration necessary to trigger aggregation is absent.

## Discussion

In many studies related to protein aggregation it has been shown that the secondary structure thermodynamic stability and *in vitro* fibril formation propensity are strongly related [13], [27], [28], [29], [30]. Temperature, pH and denaturants, such as urea and guanidinium hydrochloride, at suitable concentrations may promote protein conformation changes, particularly total or partial protein unfolding, leading to aggregation [20], [31], [32], [33]. The aggregation mechanism and kinetics as well as the morphology of the final products have been shown to depend on the specific unfolded intermediate structure [34], [35], [36], [37]. For instance, *Khurana et al.* [29] studied the aggregation of a recombinant non-amyloidogenic light chain variant (stable in physiological conditions) induced by pH, and found that amorphous aggregates were obtained at pH between 3 and 4, while at pH 2 fibrillar structures were formed. In another study [9], the morphology of the aggregates obtained *in vitro* by a single variant was significantly affected by the chemical composition of biologically relevant lipid-derived aldehydes added into the system. Also in this case, aggregation occurred via protein secondary structure changes. These examples clearly underlined the important role played by the protein secondary structure in the delicate balance between electrostatic and hydrophobic interactions responsible for the protein stability [38].

Another physical factor significantly affecting the stability behavior of the light chain is the protein native oligomeric state, i.e., monomer, dimer, and their equilibrium state. In the case of an amyloidogenic variant a single amino acid mutation located at the dimer interface could cause a significant difference in the dimer stability, shifting the equilibrium versus the monomer and promoting aggregation [39]. This finding is supported by the fact that fibril formation kinetics was accelerated when monomer-dimer equilibrium was shifted versus monomer by decreasing the total protein concentration or by denaturant addition [13], [20], [40]. In the proposed aggregation schemes, fibrillation proceeded only via monomer addition, while dimers were responsible for the off-pathway amorphous oligomers observed together with amyloid fibrils.

The results shown in this work indicate that the light chain dimer is very stable in physiological conditions and dimer denaturation is a necessary but not sufficient condition to induce aggregation. This follows from our observation that protein denaturation either by shifting the pH from 7.4 to 2 or by adding GuHCl at physiological pH was not resulting in any instability. Instead, aggregation was observed at low pH when salt was added at large concentrations or at physiological pH when the solution was heated to the protein *T_m_*. The results indicate that a specific configuration change is necessary to induce aggregation in globular proteins.

From a kinetic point of view, we observed that when aggregation occurs it starts immediately after incubation without any induction period, as indicated by DLS data (Figures 3 and 5A). The aggregation is accompanied by structural rearrangements which decrease the solvent exposure of tryptophan residues (Figure 5C) and increase the protein β-sheet content (CD data in Figure 5D). Such increase in the β-sheet content in the aggregates compared to the native state is confirmed by FTIR analysis (Figure 7). Despite such increase, the aggregates retain a large amount of random coil, α-helix and loops structures and do not bind ThT or Congo Red dye as amyloid fibrils do. AFM confirms the absence of fibrils and shows the presence of amorphous spherical aggregates (Figure 6). Moreover, the addition of such amorphous aggregates in native proteins is unable to induce aggregation in physiological conditions, most probably because they cannot induce the necessary conformational change in the native fold protein. At least for the reference reaction (run 9 of Table 1) in FFF and SEC chromatograms, the decrease in the dimer peak was not accompanied by the increase in the monomer peak, indicating that aggregation was not occurring via monomer formation. The lack of monomer formation and the absence of fibrils suggest that the monomer is the only repetitive unit present in the pathway to amyloid fibrils, while dimers are responsible for amorphous aggregates.

**Figure 8:**
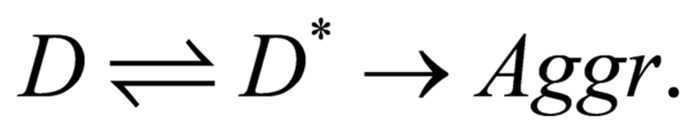
Scheme of aggregation mechanism. D* represents the salt-induced or temperature-induced intermediate dimer prone to aggregate.

Considering the experimental evidences previously discussed, we proposed the reaction scheme shown in Figure 8: under suitable conditions the native dimer is partially denatured into a reactive species D*, which may be in equilibrium with the native dimer *D*. D* has suitable conformational characteristics to aggregate further irreversibly into larger amorphous aggregates.

Of particular interest is the fact that one of the few conditions where aggregation was observed is the addition of salt at low pH. Salt-induced aggregation at low pH has been observed also for a complete monoclonal immunoglobulin IgG2: at pH 3 with NaCl in the concentration range 0.1–0.5 M, partial unfolding of the antibodies followed by reversible aggregation to oligomers was observed [41]. The effect of the salt on the stability of the light chain dimer and of the intact immunoglobulin can be explained by a combination of several factors. First of all, salt ions at low salt concentration screen the repulsive electrostatic interactions between proteins, as described by the DLVO (Derjaguin-Landau-Vervey-Overbeek) theory, which quantifies the mean-field interaction potential between two approaching particles considering only Van der Waals and non-specific electrostatic interactions [42]. Second, at the high concentrations considered in this work (0.15–0.45 M), additional, anion binding effects must be considered, which may change the protein surface chemistry and therefore the protein secondary structure, inducing a conformation more prone to aggregate. Further, salt-protein preferential exclusion may lead to salting-out effect, resulting in protein precipitation [43]. Unlike unspecific salt screening effects, such effects are strongly ion specific. In this work only anions have been found to affect aggregation kinetics, while the investigated monovalent cations (Na and K) do not have any effect. The specific anion effect on several biological phenomena has been widely reported in the literature and often compared to the so-called Hofmeister series [44] based on anion capacity to affect protein stability, protein precipitation and water structure. Despite the large use of the Hofmeister series, its molecular origin is still under debate [42]. When compared at the same concentration and similar ionic strength, the order of the anions in accelerating *in vitro* light chain aggregation found in this work is: SO_4_^−^>Cl^−^>H_2_PO_4_^−^, somehow in disagreement with the Hofmeister order: SO_4_^−^>H_2_PO_4_^−^>Cl^−^ [42].

The peculiar effect of the sulfate anion in promoting aggregation has also an *in vivo* biologically relevance due to the involvement of sulfonated glycosaminoglycans (GAGs) in amyloid fibril formation of several proteins (ex. light-chain [21], [45], [46], [47], β_2_-microglobulin [48], transthyretin [49], human muscle acylphosphatase [50], gelsolin [51]). Despite the large number of reported evidences, a clear explanation of the mechanism by which GAGs and sulfate anion promote aggregation is still lacking. In this work, we show that not only fibrillation but also aggregation into amorphous aggregates is accelerated by the sulfate anion. The peculiar effect of the sulfate may be explained considering several properties of bivalent anions: 1) at a given concentration, the screening of electrostatic repulsion of a bivalent anion is larger with respect to monovalent anions; 2) a bivalent anion can act as a bridge between two positively charged proteins favoring their aggregation [52]; 3) according to the Hofmeister series, sulfate is the most kosmotropic anion with the largest propensity to induce salting-out effect without changing the native structure; 4) the bivalency promotes a more effective anion binding, which may significantly changes intramolecular interactions, stabilizing an unfolded intermediate with respect to the native state. The experimental results indicate that, at least for the dimer investigated in this work, at low pH the last one of the above effects is predominant: sulfate anion binding induces a disruption of the β–sheet structure of the immunoglobulin variant into a more disordered structure, which apparently exhibits larger propensity to aggregate. It is worth noting that at physiological pH the protein has a lower net positive charge and anion binding is significantly reduced. Indeed, no aggregation was observed at physiological pH, even in presence of Na_2_SO_4_ or GuHCl.

### Concluding remarks

In this work, a non-amyloidogenic light chain dimer has been expressed in human myeloma cell line U266 adapted to low serum media, and its *in vitro* aggregation behavior has been investigated. Moreover, the light chain sequence has been characterized in order to allow comparative analysis with other studies describing the features of amyloidogenic light chains with a known sequence.

It is found that the dimer is very stable in physiological conditions and aggregation is observed only when specific denaturing conditions are applied.

Aggregation starts immediately without any lag phase or nucleation process and forms spherical, amorphous aggregates. The impossibility to obtain fibrils *in vitro* from a light chain dimer has a significant relevance also for the *in vivo* systems, suggesting that the presence of light chain monomer is fundamental for the formation of amyloid fibrils, while dimers are responsible for oligomers or amorphous deposits.

It is found that at low pH the salt anion has a significant specific effect on protein aggregation kinetics according to the following order: SO_4_^−^≫Cl^−^>H_2_PO_4_^−^. In particular, the sulfate anion accelerates aggregation by inducing protein secondary structure change.

## Materials and Methods

### Cell growth, protein purification and characterization

The IgE *λ* light chain was produced from the human mammalian cell line U266 obtained from a 53-years-old patient suffering from multiple myeloma [14]. Cells were received from the institute “Mario Negri” (Milan, Italy) in RPMI-1640 medium (Sigma-Aldrich, Steinheim, DE) with addition of 10% fetal bovine serum (FBS) (PAN Biotech, München, DE) and 0.1% Penicillin-Streptomycin Solution (Sigma-Aldrich, Steinheim, DE) to avoid bacterial contaminations. The cells were adapted from the 10% FBS medium to a 1% FBS medium to simplify the purification step.

The collected supernatant containing the desired product was concentrated 10-fold using a Sartoflow® Slice 200 Benchtop Crossflow system with 10 kDa cut-off membrane (Sartorius GmbH, Göttingen, DE). The concentrated cell culture supernatant was then prepared for cation exchange chromatography by three-fold dilution with deionized water for decreasing the ionic strength and by adjusting the pH to a value in the range 5.0–5.5 using glacial acetic acid (Carbo Erba Reagents, Rodano, Italy). The solution was then filtered using the Sartoflow® Slice 200 Benchtop Crossflow system with 200 nm cut-off membrane.

The clarified cell culture supernatant was then purified by combining cation exchange chromatography with size exclusion chromatography (SEC). In principle, pure protein can be obtained using a SEC column alone. However, since the SEC technique can operate only at a relatively low flow rate and only a limited amount of material can be loaded, it has extremely low productivity. Therefore, we have developed a cation-exchange process to purify and simultaneously concentrate the IgE λ light chain fragment. All preparative chromatography steps were operated on AKTA equipment (GE Healthcare, Uppsala, SE). The cation-exchange step was carried out using Poros HS50 (Applied Biosystems, Hercules, CA, USA), packed into a KronLab TAC 15/125G0-SR column with 1.5 cm inner diameter and 7.6 cm length. The chromatography method included a loading step at 10 mL/min (340 cm/h) and a linear gradient elution step from 0–100% buffer B in 10 min at 9 mL/min (305 cm/h). As mobile phases, 25 mM acetate buffer (pH 5.0, buffer A) and 25 mM acetate buffer containing 1 M NaCl (pH 5.0, buffer B) were used. The gradient elution was fractionated and the purity of the fractions was determined by analytical SEC on a HP Agilent Series 1100 equipped with a Superdex 75 10/300 GL column (GE Healthcare, Uppsala, SE). The fractions with the largest content of λ light chains were pooled, concentrated and injected into a HiLoad™ 26/60 Superdex™ 75 prep grade column (GE Healthcare, Uppsala, SE). The fractions with the largest content of *λ* light chain were washed four times with 25 mM PBS (pH 7.4, 1 g/L NaN_3_) and concentrated to reach a final concentration of 1 g/L and stored in refrigeration. With this process, from 6 litres of supernatant we can obtain 15 mg of λ chain with a purity of 97%, where bovine serum albumin is the main impurity. Three batch productions indicated good reproducibility of the developed purification process.

The protein concentration was evaluated using a Pierce® BCA Protein Assay Kit (Thermo scientific, Rockford, IL, USA) and UV absorbance at 280 nm.

The protein purity was assessed by SDS-PAGE with silver staining and Western blot analysis. For the latter, the monoclonal anti-human lambda light chain (bound and free) antibody produced in mouse (Sigma-Aldrich, Steinheim, DE) and the HRP-linked anti-alpha microglobulin antibody were used as primary and secondary antibody, respectively.

To measure protein charge properties, a protein stock solution sample was loaded into an IEF gel (pH 3–10) and run in a PhastSystem™ instrument (GE Healthcare, Buckinghamshire, UK).

### Mass Spectrometry (MS)

The mass spectrometric analysis was performed on Bruker's ESI-Qq-TOF (Bruker, Billerica, MA, USA). The protein samples at pH 2.7 and 7.4 were ionized by a nano-spray source (Advion's NanoMate, Ithaca, NY, USA). The highly protonated mass spectra were then deconvoluted by the MaxEnt aglorithm to provide singly-charged molecular mass (MH+).

### Circular dichroism (CD)

Circular dichroism (CD) spectra were measured using a Jasco-815 CD spectrophotometer (Jasco, Easton, MD, USA). Far-UV CD spectra of 0.3 g/L protein solutions were recorded from 260 to 190 nm with the temperature of the cell holder controlled at 20°C. A quartz cuvette with 0.1 cm path length was used. Spectra obtained after buffer subtraction were corrected for protein concentration and smoothed using the Savitsky-Golay function.

Protein thermal stability was evaluated by recording the spectra at several temperatures and monitoring the change in mean residue ellipticity at 196 nm. The temperature was increased from 20°C to 80°C in 5°C steps. The solution was equilibrated at each temperature for 10 min before measuring.

The fraction of unfolded conformation (*F_u_*) was obtained assuming a two-state folding mechanism according to Equation 1:
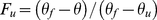
(1)where 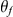 and 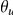 represent the mean residue ellipticity values characteristic of folded and fully unfolded protein. From the sigmoidal curve-fit to experimental data the melting temperature (*T_m_*) was evaluated.

### Intrinsic Tryptophan Fluorescence (Trp) and 8-anilino-1-naphthalenesulfonic acid (ANS) binding

Intrinsic Tryptophan Fluorescence (Trp) measurements were performed on a Varian Cary Eclipse Fluorescence Spectrophotometer (Varian, Palo Alto, CA, USA). Intrinsic tryptophan fluorescence analysis was performed exciting the sample at 295 nm and collecting emission spectra between 305 and 450 nm. The effect of denaturant on conformational stability was investigated by incubating 0.8 g/L light-chain solutions in 25 mM PBS buffer at pH 7.4 with 0.15 M NaCl and with guanidine hydrochloride (GuHCl) in the concentration range from 0 to 5 M at 25°C for 40 min. Changes in protein structure were estimated by fluorescence intensity values assuming a two-state folding mechanism according to Equation 1. From the sigmoidal curve-fit to experimental data the midpoint of the unfolding transition was evaluated (*C_m_*).

8-anilino-1-naphthalenesulfonic acid (ANS) binding experiments were performed using an EnSpire 2300 Multilabel Plate Reader (Perkin Elmer, Boston, MA, USA). Emission spectra of 0.3 g/L light chain solution in several buffers with 25 µM ANS were collected at 20°C between 420 and 600 nm using 380 nm as excitation wavelength.

### Dynamic Light Scattering (DLS)

A 10 g/L light chain stock solution was diluted to the desired concentration (0.3–1 g/L) by the desired investigated buffer solutions. DLS measurements were performed on-line using a Zetasizer Nano (Malvern, Malvern Worsc, UK). All samples were filtered with a 0.02 µm cut-off, Anotop 10 syringe filter (Whatman, Kent, UK) immediately before the experiment.

### Size Exclusion Chromatography (SEC)

The size exclusion chromatography (SEC) technique was performed with a Superdex 75 10/300 GL, 10 mm×300 mm size-exclusion column (GE Healthcare, Uppsala, SE) on a Agilent 1200 series HPLC unit (Santa Clara, CA, USA). Each sample was eluted for 60 min at a constant flow rate of 0.5 mL/min using as mobile phase a 100 mM Na_2_SO_4_, 25 mM Na_2_HPO_4_ solution at pH 7.4, filtered with a 0.45 µm cut-off, Durapore membrane filter (Millipore, Billerica, MA, USA). The UV absorbance peaks were detected at 280 nm and 220 nm.

### Field Flow Fractionation (FFF)

The asymmetrical Field Flow Fractionation (FFF) assay was performed using a AF4 Eclipse 3+ (Wyatt, Dernbach, DE), coupled with a 1200 Series isocratic pump from Agilent (Santa Clara, CA, USA). A 275 mm LC channel for aqueous solvents was used for Eclipse 3, with a trapezoidal spacer (350 µm thick, 26.5 cm long) and a Nadir reg. cellulose membrane with 1 kDa cut-off at the bottom (Wyatt, Dernbach, DE). The detector flow was set constant at 1 mL/min, and a step gradient of cross flow from 5 mL/min to 0 mL/min was applied after 30 min. 20 mM HCl buffer solution at pH 2.0 was used as mobile phase, after filtration through a 0.1 µm cut-off, Durapore membrane filter (Millipore, Billerica, MA, USA). 25 µL of sample were injected at desired time interval.

### Atomic Force Microscope (AFM)

10 µL of 30 fold diluted samples were spotted on a freshly cleaved mica surface for 30 seconds before washing with Millipore water to remove unattached material and gently drying under nitrogen flux. Samples were imaged at room temperature by a Nanoscope IIIa (Digital Instrument, USA) operating in tapping mode. Scan rate of 0.8 Hz and antimony doped silicon cantilevers with resonance frequency in the range 325–382 kHz and tip radius of 8 nm (Veeco, Plainview, NY, USA) were used.

### Fourier Transform Infrared Spectroscopy (FTIR)

Hydrated thin film attenuated total reflectance fourier transform infrared spectroscopy (ATR-FTIR) spectra were acquired on a Nicolet Nexus 870 FTIR ESP instrument equipped with a ATR Nicolet Omni-Sampler device (Nicolet, Madison, WI, USA). Aliquots of 10 µL were spotted on the crystal surface and let drying under nitrogen flux. The spectra were collected in the wavelength range from 1700 to 1600 cm^−1^ at 1 cm^−1^ resolution and smoothed using the Savitsky-Golay function after buffer subtraction.

## Supporting Information

Text S1 Characterization of the IgE λ light chain sequence in U266 cells, aggregation stability of light chain under several conditions followed by dynamic light scattering, and Thioflavin T (ThT) and Congo Red binding assay.
(DOC)

Figure S1 U266 derived IgE λ light chain nucleotide sequence. Black: variable region; Gray: junction region; underscore: constant region.
(TIF)

Figure S2 Deduced amino acid sequence of the IGLV2-8 derived U266 λ light chain variable region. Amino acid changes from the germline donor, IGVL2-8, are highlighted. FR: framework region; CDR: complementarity determining region.
(TIF)

Figure S3 Time evolution of light scattering intensity for run 1 (×), run 7 (*), run 9 (Δ), run 11 (•), run 12 (◊), run 13 (□) and run 14 (○) in Table 1.
(TIF)

Figure S4 Time evolution of ThT fluorescence values (♦) and DLS intensity (○) under the conditions of Run 8 in Table 1. Insert represents ThT values at longer incubation times.
(TIF)

Figure S5 Congo Red spectrum obtained by the difference between the samples and the blank solution: light chain aggregates after 10 h incubation under the conditions of Run 8 in Table 1 (–); stable light chain solution at pH 7.4 (…); insulin fibrils (―).
(TIF)
