# Supplementary material for: In Vitro Aggregation Behavior of a Non-Amyloidogenic λ Light Chain Dimer Deriving from U266 Multiple Myeloma Cells
Source: PLoS One. 2012 Mar 14;7(3):e33372. doi: 10.1371/journal.pone.0033372 (PMC3303827; doi:10.1371/journal.pone.0033372)
Supplement: Text S1 — Characterization of the IgE λ light chain sequence in U266 cells, aggregation stability of light chain under several conditions followed by dynamic light scattering, and Thioflavin T (ThT) and Congo Red binding assay. (DOC) [file pone.0033372.s001.doc]

**Supporting Information Text**

***In vitro* aggregation behavior of a non-amyloidogenic λ light chain dimer deriving from U266 multiple myeloma cells**

Paolo Arosio, Marta Owczarz, Thomas Müller-Späth, Paola Rognoni,^§^ Marten Beeg,

Hua Wu, Mario Salmona,^†^ Massimo Morbidelli*

Institute for Chemical and Bioengineering, Department of Chemistry and Applied Biosciences, ETH Zurich, 8093 Zurich, Switzerland

^†^Department of Molecular Biochemistry and Pharmacology, Istituto di Ricerche Farmacologiche ‘‘Mario Negri’’, Milan, Italy

^§^ Amyloid Research and Treatment Center, Foundation IRCCS Policlinico San Matteo, University of Pavia, Italy

**Characterization of the IgE λ light chain in U266 cells**.

Total RNA was extracted from 10^7^ U266 cells. The IgE λ light chain nucleotide sequence was cloned by a universal inverse-PCR strategy.[^1^](#_ENREF_1) Briefly, primers specific for the 5’ (λ-C_LA_) and 3’ (λ-C_LB_) regions of the constant region of the λ light chain were used to deduce the Vλ chain sequence for specific variable region primer design. The PCR primers were characterized by the following sequences: λ-C_LA_: 5’-AGTGTGGCCTTGTTGGCTTG-3’ (from codon +132 to +126), λ-C_LB_: 5’-GTCACGCATGAAGGGAGCAC-3’ (from codon +196 to +204). In order to obtain the original full-length light chain sequence (variable region + constant region, from codon +1 to +216), standard RT-PCR was employed using the same RNA, 5’ clone-specific primer (5’-CAGTCTGCCCTGACTCAGCCT-3’, from codon +1 to +7 of variable region) and a 3’ universal Cλ carboxy-terminal primer, corresponding to the last amino acids of the constant region (5’-TGAACATTCTGTAGGGGCCAC-3’, from codon +210 to +216). The PCR fragment was ligated into a cloning vector and amplified. After recombinant plasmid purification, insert was sequenced (Figure S1).

To determine the IgE λ light chain presumed germline, sequence alignment was made with the current releases of EMBL-GenBank, V-BASE (V BASE Sequence Directory, MRC Centre for Protein Engineering, Cambridge, UK) and IMGT sequence directories. The sequence showed the highest homology with the IGVL2-8 germline (Figure S2).

**Aggregation stability of light chain under several conditions**

The aggregation stability of the light chain was investigated under all the conditions reported in Table 1 by dynamic light scattering (DLS). The stability results have been summarized in Table 1. In Figure S3 the time evolution of the scattered intensity for all the conditions not reported in the paper is shown.

**Thioflavin T assay (ThT) and Congo Red binding**

For the reference aggregating condition (Run 8 in Table 1), the aggregation kinetics and aggregate morphology have been investigated more in details. Off-line Thioflavin T assays were measured during aggregation on a Varian Cary Eclipse Fluorescence Spectrophotometer (Varian, Palo Alto, CA, USA). Samples of 10 μL were diluted into 990 μL 25 mM PBS buffer at pH 7.4 with 10 μM Thioflavin T (Acros Organics, Geel, Belgium), as reported by *Raffen et al.*[^2^](#_ENREF_2) The measurements were performed at 25^o^C with excitation at 450 nm and emission at 485 nm. The time evolution of ThT fluorescence as well as DLS intensity values is shown in Figure S4: despite the large increase of the light scattering intensity in the first 10 hours, the increase of ThT absolute fluorescence values is small even after three weeks of incubation, indicating a low increase in the β-sheet content.

The result was confirmed by Congo Red assay, performed recording the UV spectrum between 400 and 700 nm on an EnSpire 2300 Multilabel Plate Reader (Perkin Elmer, Boston, MA, USA). 7.5 μL of protein aggregate solution after 10 h of incubation were added to a blank solution of 20 μM Congo Red (Fisher Scientific, Loughborough, UK) in 25 mM PBS buffer at pH 7.4 and left incubated for 30 minutes at room temperature. In presence of amyloid fibrils the spectrum obtained by the difference between the sample and the blank solution should present a maximum at 540 nm.[^3^](#_ENREF_3)

A stable light chain solution at pH 7.4 and a 5 g/L insulin fibrils solution were analyzed as reference for non-amyloidogenic and amyloidogenic structures, respectively. In Figure S5 it can be seen how the spectra of the light chain aggregates and of the stable light chain solution are similar and show no maximum; on the other hand, the spectrum of insulin fibrils shows a clear maximum at 541 nm. The results confirm the absence of fibrillar, amyloidogenic structures in the light chain aggregates.

1. Perfetti, V., Sassano, M., Ubbiali, P., Vignarelli, M. C., Arbustini, E., Corti, A. & Merlini, G. (1996). Inverse polymerase chain reaction for cloning complete human immunoglobulin variable regions and leaders conserving the original sequence. *Analytical Biochemistry* **239**, 107-109.

2. Raffen, R., Dieckman, L. J., Szpunar, M., Wunschl, C., Pokkuluri, P. R., Dave, P., Stevens, P. W., Cai, X. Y., Schiffer, M. & Stevens, F. J. (1999). Physicochemical consequences of amino acid variations that contribute to fibril formation by immunoglobulin light chains. *Protein Science* **8**, 509-517.

3. Nilsson, M. R. (2004). Techniques to study amyloid fibril formation in vitro. *Methods* **34**, 151-160.
