# Supplementary figures and images for: In Vitro Aggregation Behavior of a Non-Amyloidogenic λ Light Chain Dimer Deriving from U266 Multiple Myeloma Cells
Source: PLoS One. 2012 Mar 14;7(3):e33372. doi: 10.1371/journal.pone.0033372 (PMC3303827; doi:10.1371/journal.pone.0033372)

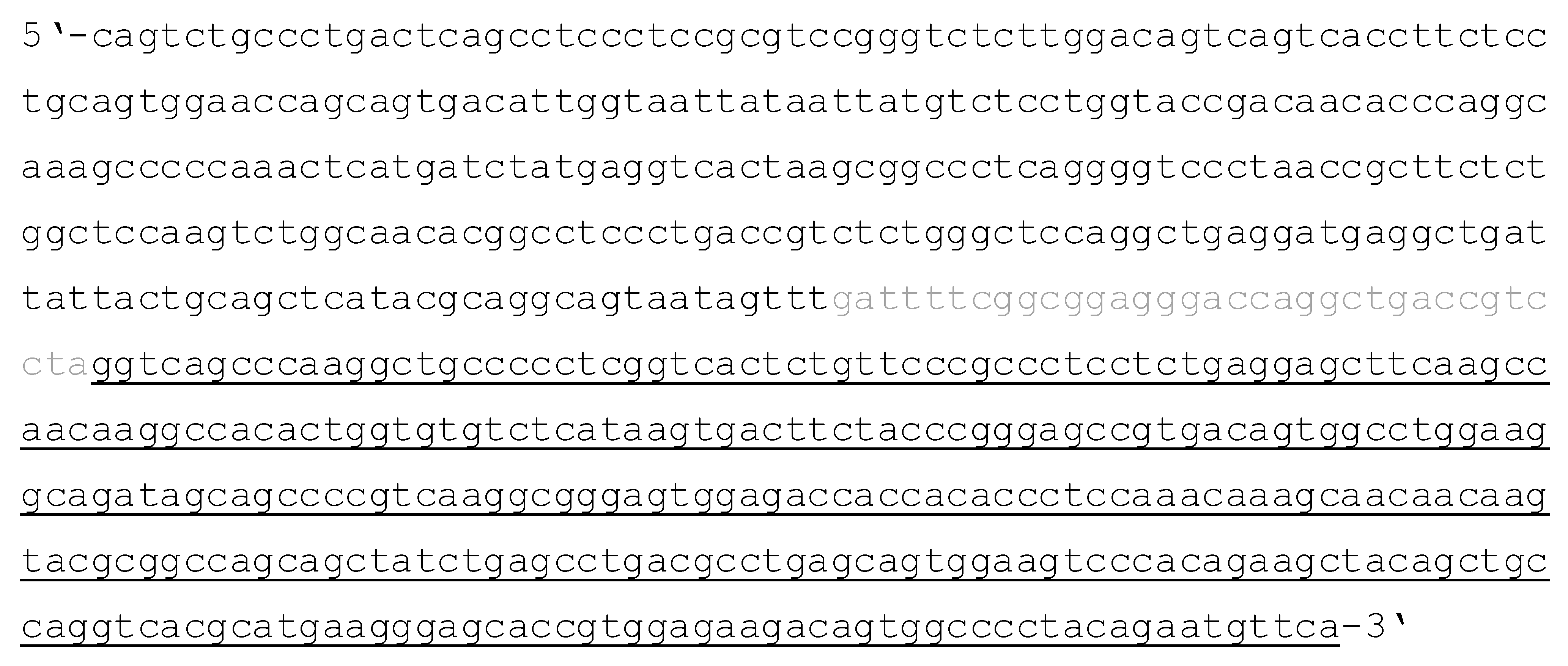

Supplement: Figure S1 — U266 derived IgE λ light chain nucleotide sequence. Black: variable region; Gray: junction region; underscore: constant region. (TIF) [file pone.0033372.s002.tif]

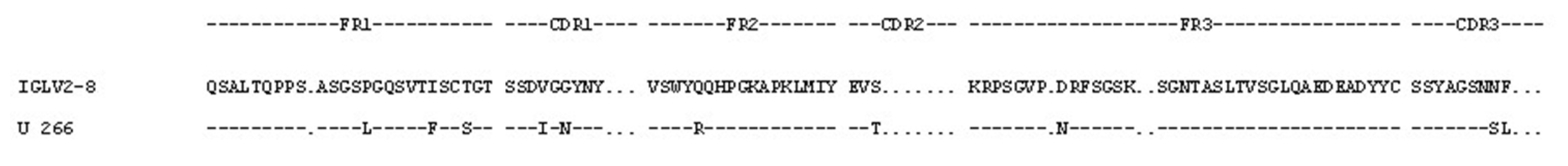

Supplement: Figure S2 — Deduced amino acid sequence of the IGLV2-8 derived U266 λ light chain variable region. Amino acid changes from the germline donor, IGVL2-8, are highlighted. FR: framework region; CDR: complementarity determining region. (TIF) [file pone.0033372.s003.tif]

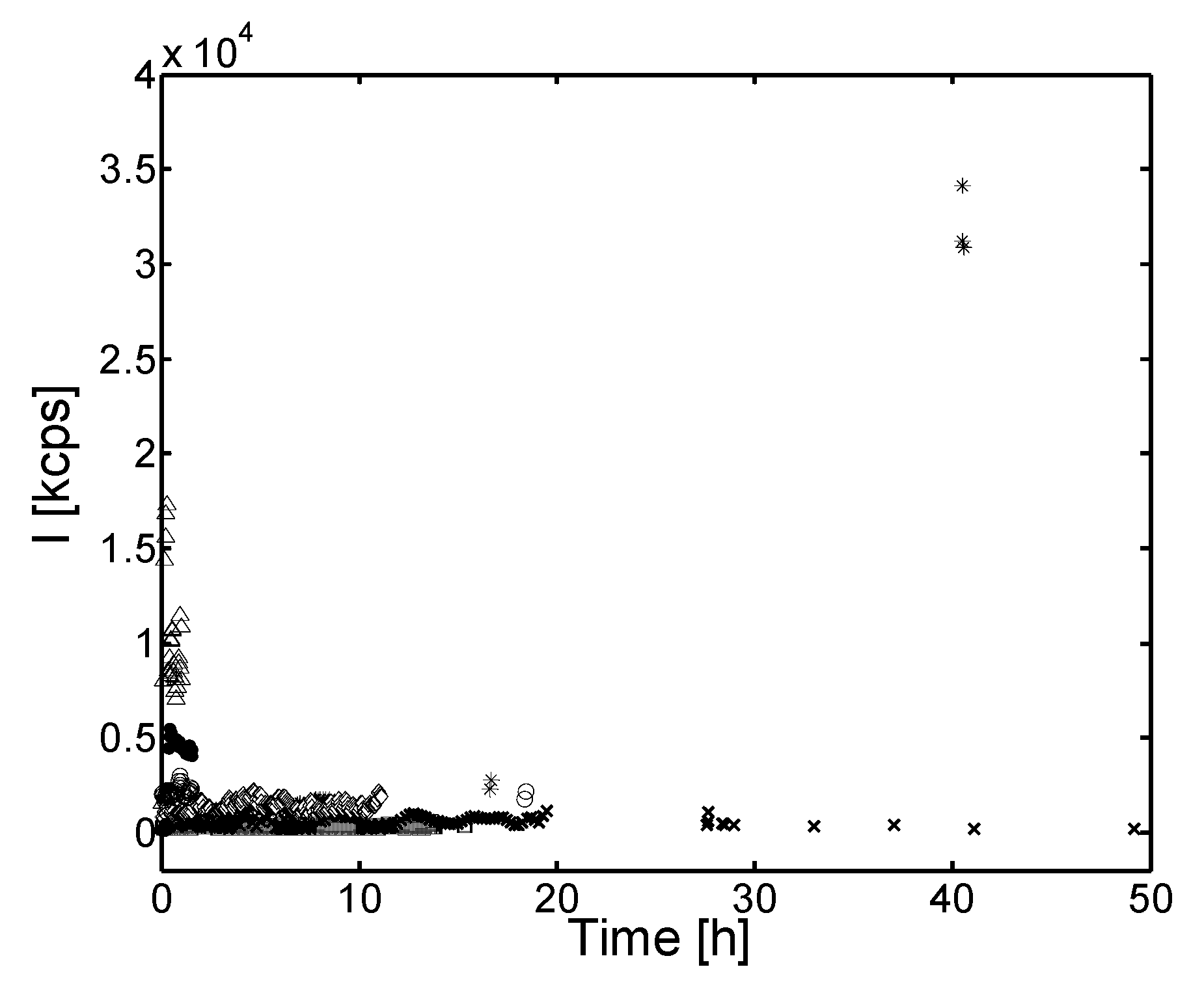

Supplement: Figure S3 — Time evolution of light scattering intensity for run 1 (×), run 7 (*), run 9 (Δ), run 11 (•), run 12 (◊), run 13 (□) and run 14 (○) in Table 1. (TIF) [file pone.0033372.s004.tif]

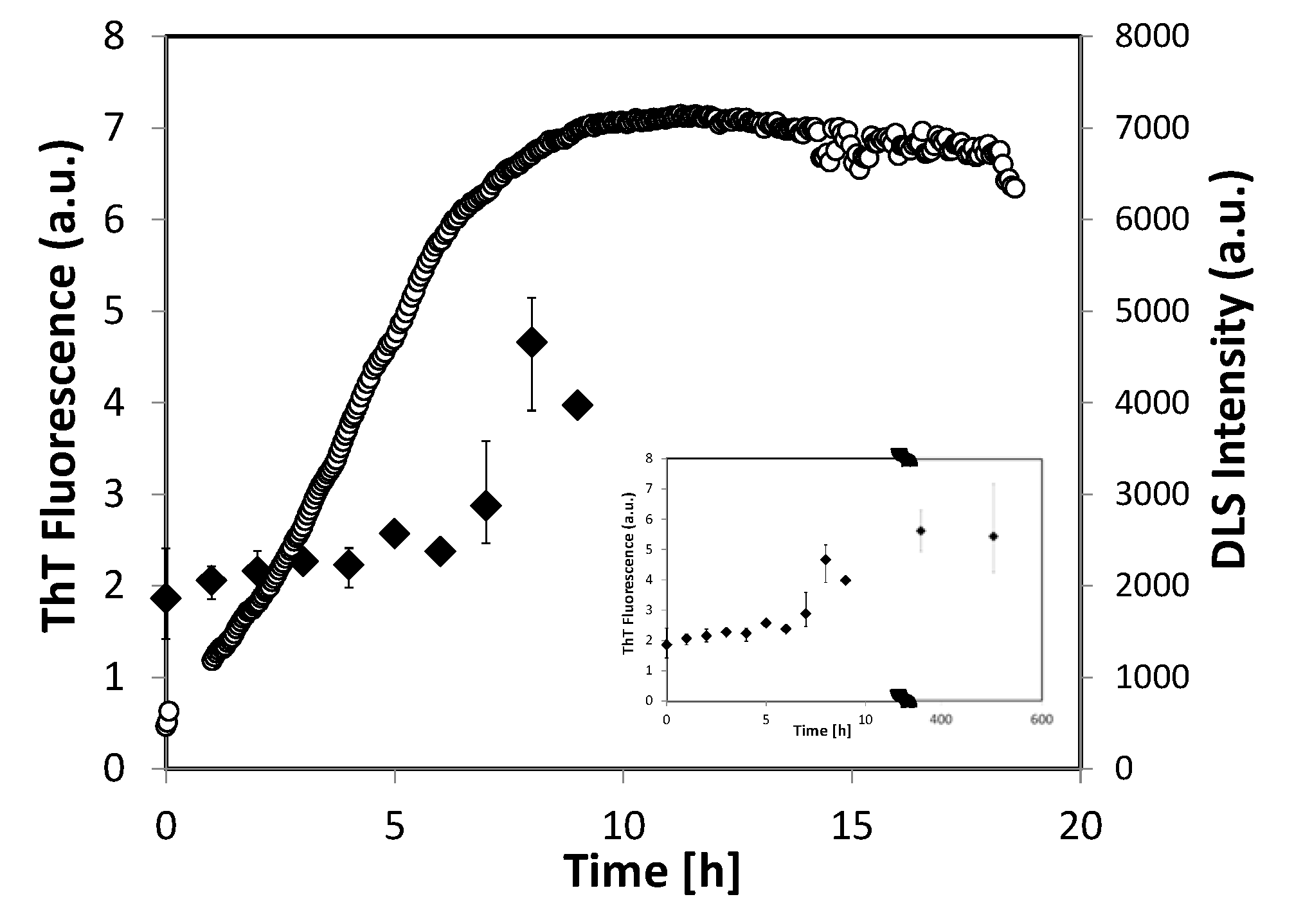

Supplement: Figure S4 — Time evolution of ThT fluorescence values (♦) and DLS intensity (○) under the conditions of Run 8 in Table 1. Insert represents ThT values at longer incubation times. (TIF) [file pone.0033372.s005.tif]

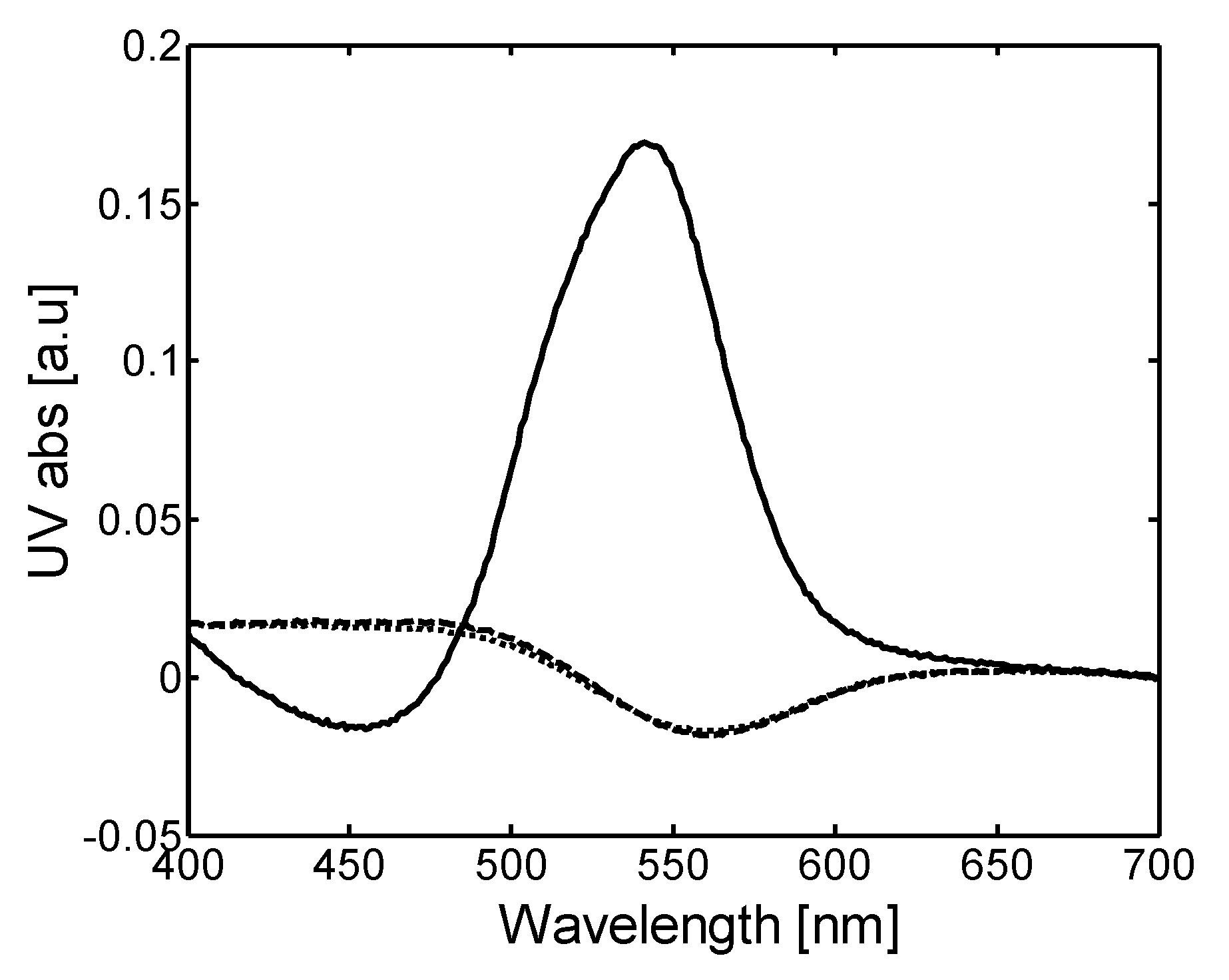

Supplement: Figure S5 — Congo Red spectrum obtained by the difference between the samples and the blank solution: light chain aggregates after 10 h incubation under the conditions of Run 8 in Table 1 (–); stable light chain solution at pH 7.4 (…); insulin fibrils (―). (TIF) [file pone.0033372.s006.tif]
